# A first country-wide review of Diabetes Mellitus care in Bhutan: time to do better

**DOI:** 10.1186/s12913-015-1026-6

**Published:** 2015-09-17

**Authors:** Kinley Zam, Ajay MV Kumar, Shanta Achanta, Prashant Bhat, Balaji Naik, Kado Zangpo, Tandin Dorji, Yeshey Wangdi, Rony Zachariah

**Affiliations:** Policy and Planning Division, Ministry of Health, Thimphu, Bhutan; International Union Against Tuberculosis and Lung Disease, South-East Asia Regional Office, New Delhi, India; WHO Country Office for India, New Delhi, India; Department of Public Health, Ministry of Health, Thimphu, Bhutan; National Diabetes Control Programme, Department of Medical Services, Ministry of Health, Thimphu, Bhutan; Medecins Sans Frontieres, Brussels Operational Center(Operational Research), Luxembourg City, Luxembourg

## Abstract

**Background:**

There is an increasing trend of non-communicable diseases in Bhutan including Diabetes Mellitus (DM). To address this problem, a National Diabetes Control Programme was launched in 1996. There is anecdotal evidence that many patients do not visit the DM clinics regularly, but owing to lack of cohort monitoring, the magnitude of such attrition from care is unknown. Knowledge of the extent of this problem will provide a realistic assessment of the situation on the ground and would be helpful to initiate corrective actions. In this first country-wide audit, we thus aimed to determine among type 2 DM patients registered for care the i) pre-treatment attrition ii) one-year programme outcomes including retention in care, died and Lost–to-follow-up (LTFU, defined as not having visited the clinic at least once within a year of registration) iii) factors associated with attrition from care (death + LTFU) and iv) quality of follow-up care, measured by adherence to recommended patient-monitoring protocols including glycaemic control.

**Methods:**

A retrospective cohort study involving a review of records routinely maintained under the National Diabetes Control Programme. All type 2 DM patients registered between 1st January and 31st December 2012 in 18 district hospitals of Bhutan were included. Glycaemic control was defined as glycosylated haemoglobin of <7 % or [Fasting Blood Sugar of <130 mg/dl and, Post-prandial Blood Sugar of <180 mg/dl].

**Results:**

Of 350 registered DM patients (52 % female, median age 55 years), 63(18 %) were LTFU before treatment initiation (pre-treatment attrition). Of the remaining 287 individuals who started treatment, 226(79 %) were retained in care while 61(21 %) either died or were LTFU. Glycaemic control was achieved in 85(38 %) patients retained in care. Between 7 and 98 % of monitoring parameters had missing data.

**Conclusion:**

Nearly one-third of DM patients were LTFU and there were short comings in monitoring. Qualitative research is urgently needed to find out the reasons for high attrition. Given the high political commitment by the Royal Government of Bhutan, the findings provide ample grounds for instituting corrective measures and propelling DM care further. It is time to do better!

## Background

Diabetes Mellitus (DM) is currently a major public health problem associated with lifelong complications and massive health care expenditure. According to the International Diabetes Federation, in 2014, the number of people with DM stood at 387 million (nearly half were undiagnosed), with about 75 million in South East Asia alone. This figure is expected to increase to 592 million by 2035. For the same year, the International Diabetes Federation estimated total health expenditure on diabetes to be about 612 billion United States Dollars [1].

Type 2 DM accounts for more than 90 % of all DM and is a major risk factor for cardiovascular disease and mortality [2]. However, early diagnosis and prompt care to achieve glycaemic control have been shown to reduce the risk of complications and improve the quality of life [3]. In most developing countries, DM management is characterized by sub-standard care and complications are often not prevented, recognized or treated. Unstructured and unmonitored clinical care is the norm and there is little regular or reliable information about incident and prevalent cases, treatment outcomes, morbidity and mortality [3].

Bhutan is a small landlocked country in South-East Asia with a population of 745,153 [4]. As signatory to the Alma Ata Declaration of 1978, Bhutan adopted the primary health care approach to offering health care. Health care services are provided through a three tiered system with ascending hierarchy. Basic Health Units and outreach clinics at community level, district hospitals at the secondary level and regional and national referral hospitals at the tertiary level. As enshrined in the constitution, the state provides free basic health services to all citizens of Bhutan. The health system is predominately financed by public sources through general revenues; the private practice is limited to few established diagnostic centers in few major cities of the country.

While, Bhutan witnessed a remarkable improvement in recent years in terms of achieving the key health indicators and health-related Millennium Development Goals (MDGs), there is an increasing trend of non-communicable diseases (NCDs) including DM [5, 6]. A STEPS survey (a STEPwise approach to surveillance recommended by WHO) conducted in Bhutan 93 % of the population of the capital city of Thimphu was exposed to at least one risk factor for NCDs (tobacco use, alcohol consumption, diet and physical activity) for NCDs [7]. There are currently close to 1,500 registered diabetic patients in Thimphu alone [8].

To address this growing problem, a National Diabetes Control Programme (NDCP) was started by the Ministry of Health of Bhutan in 1996. Diabetes clinics were set up in all the 20 district hospitals across the country to manage DM. There is anecdotal evidence that many patients do not visit the DM clinics regularly and there is considerable losses-to-follow-up (LTFU) [3]. However, owing to a lack of cohort monitoring and reporting of registered DM patients, the magnitude of any such attrition from care is unknown and so too the quality of DM care being provided. Better knowledge of these parameters will help provide a realistic assessment of the situation on the ground and may catalyse corrective actions.

In this first country-wide audit, we aimed to determine among type 2 DM patients registered for care in 2012 the i) pre-treatment attrition ii) one-year programme outcomes (retained in care, died and LTFU) iii) factors associated with attrition from care (combination of deaths and LTFU) and iv) quality of follow-up care measured by assessing adherence to recommended protocols for patient monitoring including glycaemic control.

## Methods

### Design

A retrospective cohort study involving a review of records routinely maintained under the NDCP.

### Study population and study period

All type 2 DM patients newly registered from 1st January to 31st December 2012 in 18 district hospitals of Bhutan were included in the study. Two Regional Referral hospitals and one National Referral Hospital were excluded from the study due to the lack of follow-up data in these diabetes clinics. The studies was conducted between March and December 2014.

### Setting

In addition to sub-post and Basic Health Units at the primary level there are 20 District Hospitals, two Regional Referral Hospitals and one National Referral Hospital. Diabetes clinics are located at all levels starting from selected basic health units to the District and tertiary hospitals. All services are provided free-of-charge.

### Diagnosis of DM

All patients suspected of having DM are screened and managed at DM clinics. DM screening is performed by measuring random blood glucose (RBG) upon attendance at the clinic. The threshold is set at >200 mg⁄dl of blood glucose with accompanying clinical features [9]. Confirmation of type 2 DM is through two fasting plasma glucose (FPG) measurements, (both of which must be ≥126 mg⁄dl) and two hours post prandial plasma glucose (PPPG) ≥ 200 mg/dl [9]. Those with FPG between 110 and 125 mg⁄dl are further screened using an oral glucose tolerance test (OGTT). Patients with DM are also assessed for complications and co-morbidities such as hypertension (defined as a blood pressure of 140⁄90 mm Hg or above) and are managed accordingly. All patients diagnosed with DM, they are registered and offered treatment.

### Treatment of DM

DM patients are initiated on appropriate treatment including advice on dietary control and lifestyle modifications, oral hypoglycaemic drugs and/or insulin depending upon the glycaemic levels. The Ministry of Health, Bhutan provides oral medicines like Glibenclamide, Glipizide and Metformin. Insulin in form of human (soluble) insulin, Human insulin zinc suspension, human mixtard (neutral + isophane) is also available [10].

### Follow-up care protocol of DM patients

As per standard national protocol, [9] DM Patients are expected to visit the DM clinic once every month for follow-up care or as deemed necessary by the attending clinician. Weight, blood pressure measurement, FBS, PPBS and foot examination are done monthly; glycosylated haemoglobin (HbA1c) and dental examination are done quarterly; blood urea, serum creatinine and lipid profile are assessed on a six monthly basis. Patients with uncontrolled type 2 DM and complications are referred to higher centres or even countries like India.

### Data collection, definition of programme outcomes, and glycaemic control

Data related to the study objectives were extracted from the Diabetes Registry maintained in each clinic into a structured proforma. When data was not available in the registry, patient follow-up records were used to source data. The numbers of follow-up visits during the year as well as the following parameters at the latest follow-up visit were documented: HbA1C, foot examination, dental examination, and blood pressure to assess the adherence to care protocol. Treatment outcomes were censored on 31 December 2013 ensuring that all the patients in the cohort had a follow-up period of at-least one year. There are no formal definitions of programme outcomes within the national DM programme and thus for the purposes of this analysis, outcomes were categorized as follows: alive and retained in care, died and LTFU. If a patient had not visited the DM clinic at least once within a year of registration, he/she was considered to be “LTFU”. All patients who had at-least one visit were considered to be “retained in care”. Patients who died due to any cause, while on treatment were declared as ‘death’. Patients who were registered (and diagnosed as DM) but had no treatment recorded were considered to have been LTFU prior to treatment and these patients were classified as “pre-treatment attrition”.

Glycaemic control was defined using data of FBS, PPBS and HbA1c at the latest follow-up visits. If HbA1c value was available, a value of < 7 % was set as the threshold for defining glycaemic control. In case HbA1c was not available, the FPG of 70–130 mg/dl and 2 h PPPG of < 180 mg/dl were considered to indicate glycaemic control as per the recommendations of the American Diabetes Association [11].

### Data entry and analysis

The data were double entered, validated and analysed using EpiData (version 3.1 for entry and version 2.2.2.182 for analysis, EpiData Association, Odense, Denmark). Tests of proportion was done using Chi square test. The level of significance was set at 5 %.

### Ethics

Ethics approval was obtained from the Research Ethics Board of Health, Ministry of Health, Thimphu, Bhutan and the Ethics Advisory Group of International Union Against Tuberculosis and Lung Disease, Paris, France. Since the study was based on review of records with no patient interaction, ethics committees waived the need for individual patient informed consent.

## Results

The demographic and clinical characteristics of 350 registered DM patients in Diabetes clinic in 18 Districts hospital in Bhutan (2012) are shown in Table 1. The majority of patients were female, middle aged or over and either overweight or obese. About four in ten had associated hypertension and the great majority of patients were on oral hypoglycemic drugs.

**Table 1:** Socio-demographic and clinical characteristics of DM(type 2) patients registered in diabetes clinics, Bhutan

Category	Sub category	Number (%)
Total		350
Age	< 35	23 (7)
	35–44	63 (18)
	45–54	80 (23)
	55–64	103 (29)
	≥ 65	81 (23)
Sex	Male	166 (47)
	Female	183 (52)
	Unknown	1 (< 1)
Occupation	Farmer	148 (42)
	Others	86 (24)
	Unknown	52 (15)
	Civil Services	34 (10)
	Business	30 (9)
Region	East	164 (47)
	West	122 (35)
	Central	64 (18)
Body Mass Index (kg/m^2^)	< 25	82 (23)
	25.0–29.9	112 (32)
	≥ 30.0	89 (25)
	Unknown	67 (19)
Associated Hypertension	No	224 (64)
	Yes	126 (36)
Treatment regimen	Oral Hypoglycaemic	270 (77)
	Insulin	11 (3)
	Others^a^	6 (2)
	Unknown	63 (18)
Referred for complications^b^	Yes	12 (3)
	No	338 (97)

^a^medical nutrition therapy; ^b^“Referred for complication” means the act of referring patients who developed complications due to DM (nephropathy, neuropathy and retinopathy) to regional and national referral hospitals for appropriate care

**Fig. 1:**
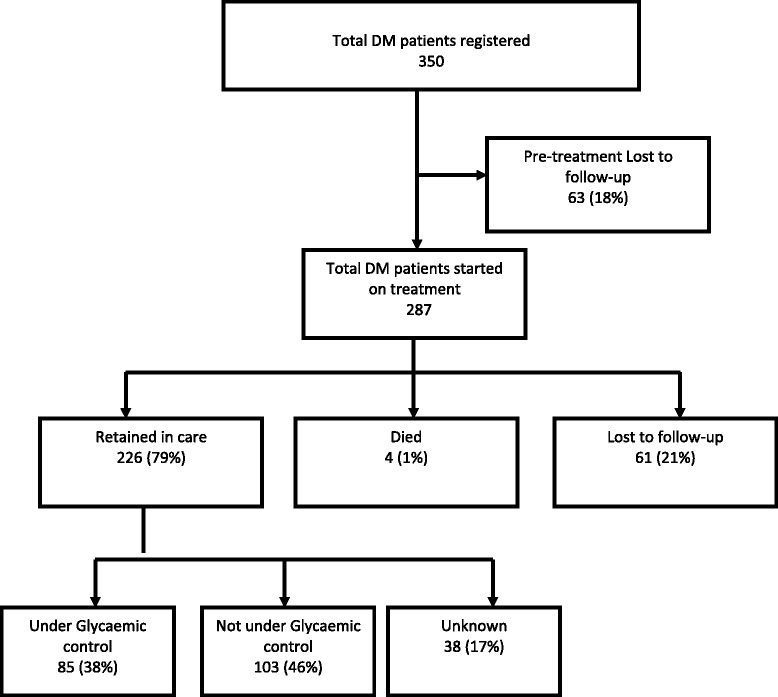
One-year programme outcomes of DM (type 2) patients registered in diabetes clinics in Bhutan

Figure 1 shows the one-year including glycemic control of patients. Of 350 registered patients, 63 (18 %) were lost to follow up before treatment initiation (pre-treatment attrition). Of the remaining 287 individuals who started treatment, 226 (79 %) were retained in care while 61 (21 %) either died or were lost-to-follow-up (on-treatment attrition). Glycaemic control was achieved in only 85 (38 %) of 226 patients retained in care (Fig. 1).

Table 2 shows factors associated with attrition. The only significant factor was geographic region.

**Table 2:** Factors associated with attrition of DM (Type2) after treatment among patients registered in diabetes clinics, Bhutan

Category	Sub category	Retained in care n (%)	Attrition n (%)	*P* value
Total (n-350)		226	124	
Age group	< 55 years	107 (79)	29 (21)	1.0
	> 55 years	119 (79)	32 (21)	
Sex	Male	105 (79)	28 (21)	0.9
	Female	121 (79)	33 (21)	
Region	West	105 (71)	42 (29)	< 0.01
	East	73 (86)	12 (14)	
	Central	48 (87)	7 (13)	
Associated Hypertension	No	132 (78)	37 (22)	0.8
	Yes	94 (80)	24 (20)	
Treatment regimen	Oral Hypoglycemics	212 (79)	58 (22)	0.51
	Insulin	10 (91)	1 (9)	
	Other	4 (67)	2 (33)	
Body Mass Index(kg/m^2^)	< 25	64 (84)	12 (16)	0.7
	25.0-29.9	84 (79)	22 (21)	
	≥ 30	65 (79)	17 (21)	
	Unknown	13 (57)	10 (43)	
Occupation	Civil Service	25 (86)	4 (14)	0.2
	Business	24 (89)	3 (11)	
	Farmer	93 (82)	21 (18)	
	Others	55 (72)	21 (28)	
	Unknown	29 (71)	1é (29)	

Table 3 shows parameters used for assessing quality of care among DM patients who were retained in care. Several of the clinical and laboratory parameters that should have been assessed were not done – the latter ranged from 2-98 %. While most patients had measurement of weight, blood pressure, FPG and PPPG, only 2 % o had their HbA1c done as per schedule.

**Table 3:** Quality of care of DM (Type 2) patients retained in care in diabetes clinics, Bhutan

Category	Sub category	Number (%)
No. of Follow up visits/year	1-3	54 (24)
	4-6	47 (21)
	7-9	73 (32)
	≥ 10	52 (23)
Weight measurement	Done	204 (90)
	Not done	22 (10)
Blood pressure measurement	Done	211 (93)
	Not done	15 (7)
Fasting blood sugar	Done	187 (83)
	Not done	39 (17)
Post prandial blood sugar	Done	192 (85)
	Not done	34 (15)
HbA1C	Done	5 (2)
	Not done	221 (98)
Foot examination	Done	133 (59)
	Not done	93 (41)
Dental examination	Done	113 (50)
	Not done	113 (50)
Blood urea	Done	108 (48)
	Not done	118 (52)
Serum creatinine	Done	108 (51)
	Not done	110 (49)
Serum triglyceride	Done	94 (42)
	Not done	132 (58)
Blood cholesterol	Done	96 (43)
	Not done	130 (57)

## Discussion

This first country-wide review of DM care in Bhutan, highlights a number of operational challenges that merit focused attention. About four-in-ten registered patients were lost to attrition with similar losses occurring both before and after initiating DM treatment. Among those retained in care, glycemic control was achieved only in one-third of all patients and considerable shortcomings were observed in routine clinical and laboratory monitoring. The findings from this review thus provide ample grounds for instituting corrective measures.

The issue of losses to follow up and achieving high levels of glycemic control are well known problems facing DM programs worldwide – both in industrialized and Low and Middle Income countries. It is thus not exclusive to Bhutan. On-treatment losses to follow up in large cohorts of DM patients in Cambodia and Kenya ranged between 32–34 % [12, 13]. In terms of glycemic control, in the United States of America and the United Kingdom, this was achieved in only 24–36 % of patients [12, 14, 15]. Similarly, in a well-resourced Non-Governmental Organization clinic in Kenya, Glycemic control was achieved in only 20 % of the study cohort [12].

The study strengths were that it was country-wide and conducted within the routine framework of care and thus likely to reflect the ground reality. It also responds to what has been expressed as a national priority in operational research. The study limitations are that we do not know the exact reasons for losses to follow up prior to, and after DM treatment initiation. This requires specific further research using qualitative research methods. In addition, missing data on clinical and laboratory parameters, made it difficult to assess, in a robust manner possible risk factors for attrition.

These limitations not-withstanding, the study raises a number of operational issues that merit consideration. First, possible reasons for attrition may include incomplete recording of patient data by DM clinic staff. This may have been aggravated by frequent changes in the clinic staff as some of them were deputed to running other busy out-patient clinics. From a patient perspective, most were poor farmers who lived in rural areas of Bhutan and transport related expenditure for follow-up visits may have posed difficulties in adhering to follow up schedules. Furthermore, until recently, DM clinics were operating only once, or twice a week and the resulting long patient waiting-times might have had a negative influence on patient acceptability. This problem should be resolved with all clinics now operating at least five days a week. Possible ways forward to improve retention in DM care would include having dedicated DM staff and in particular a data clerk, training on registration and monitoring procedures and regular supervision. Bringing in dedicated counsellors might also be important for enhancing patient empowerment and awareness for adhering to follow up.

Second, the level of glycemic control achieved during follow up was low and only 2 % of patients actually had an HbA1c test done - the most reliable parameter for assessing glycaemic control. A similar problem has been reported from other countries [12, 13]. One of the main reasons for this short-coming was unavailability of HbA1c at the peripheral DM clinics requiring patients to make out-of-pocket expenditure and travel to distantly located regional/national hospitals. To add to this problem, there have been anecdotal reports of machine breakdown and shortage of laboratory reagents. Those that present at centralized sites may thus be faced with disappointments These issues need to be looked into and the possibility of introducing decentralized access to HbA1C testing in all DM clinics or a mechanism of specimen collection and transport need to be considered.

Finally, like many of the DM programs world-wide, monitoring and evaluation is fragmented without a structured approach [3]. In our setting, case definitions for key program outcomes involving “retention” and “attrition” are yet to be defined. In addition, no system exists for feedback when patients are transferred between different DM clinics. Possible ways forward to address this important program issue include: introduction of quarterly cohort reporting for DM [12], use of electronic medical records [13, 16, 17] and routine supervision. Experiences from other countries have shown promising results with such an endeavor [11, 12].

## Conclusion

Nearly one-third of DM patients were LTFU and there were short comings in patient monitoring. Qualitative research is urgently needed to find out the reasons for high attrition. Given the high political commitment by the Royal Government of Bhutan, the findings provide ample grounds for instituting corrective measures and propelling DM care further. It is time to do better!

## Abbreviations

DM: Diabetes mellitus
NDCP: National diabetes care programme
LTFU: Loss to follow up
FPG: Fasting plasma glucose
PPPG: Post prandial plasma glucose
FBG: Fasting blood glucose
PPBG: Post prandial blood glucose
